# Antimicrobial Prescribing Practices in Small Animal Emergency and Critical Care

**DOI:** 10.3389/fvets.2020.00110

**Published:** 2020-02-28

**Authors:** Sarah N. Robbins, Robert Goggs, Guillaume Lhermie, Denise F. Lalonde-Paul, Julie Menard

**Affiliations:** ^1^Department of Clinical Sciences, College of Veterinary Medicine, Cornell University, Ithaca, NY, United States; ^2^Department of Population Medicine and Diagnostic Sciences, College of Veterinary Medicine, Cornell University, Ithaca, NY, United States; ^3^IHAP, Université de Toulouse, INRA, ENVT, Toulouse, France

**Keywords:** antibiotic, prescription, stewardship, dogs, cats, resistance

## Abstract

**Background:** Antimicrobial use contributes to emergence of antimicrobial resistance. It was hypothesized that antimicrobial prescribing behavior varies between the emergency (ER) and critical care (CC) services in a veterinary teaching hospital. This study aimed to: (i) describe antimicrobial prescribing patterns in the ER and CC services; (ii) assess adherence to stewardship principles; (iii) evaluate the prevalence of multidrug resistant (MDR) bacterial isolates.

**Methods:** Institution electronic medical records were queried for all antimicrobial prescriptions from the ER and CC services between 1/1/2017 and 12/31/2017. Prescriptions were manually reviewed, and the following data recorded: drug, dosage, duration, diagnosis, outcome, hospitalization duration, culture submission, and susceptibility results.

**Results:** There were 5,091 ER visits, of which 3,125 were not transferred to another service. Of these emergency visits, 516 (16.5%) resulted in 613 antimicrobial drug prescriptions. The most commonly prescribed drugs for the ER were amoxicillin/clavulanate (*n* = 243, 39.6%), metronidazole (*n* = 146, 23.8%), and ampicillin/sulbactam (*n* = 55, 9.0%). The most common reasons for antimicrobial prescriptions were skin disease (*n* = 227, 37.0%), gastrointestinal disease (*n* = 173, 28.2%), and respiratory disease (*n* = 50, 8.2%). For ER patients 18 cultures were submitted, equivalent to a 3.5% submission rate. The CC service managed 311 case visits for 822 patient days. Of these, 133 case visits (42.7%) resulted in 340 prescriptions. The most commonly prescribed drugs for the CC service were ampicillin/sulbactam (*n* = 103, 30.3%), enrofloxacin (*n* = 75, 22.1%), and metronidazole (*n* = 59, 17.4%). The most common reasons for antimicrobial prescriptions were gastrointestinal disease (*n* = 106, 31.2%), respiratory disease (*n* = 71, 20.9%), and sepsis (*n* = 61, 17.9%). On the CC service, 46 patients had ≥1 culture submitted, equivalent to a 34.6% submission rate. Of patients prescribed antimicrobials, 13/38 (34%) with urinary tract disease, 2/28 (7%) with pneumonia, 1/11 (9%) with canine infectious respiratory disease complex and 2/8 (25%) with feline upper respiratory infection were compliant with published guidelines.

**Conclusions:** Antimicrobial prescription was common in both ER and CC services and followed similar patterns. Adherence to published guidelines for urinary and respiratory infections was poor.

## Introduction

Epidemiological studies suggest a link between antimicrobial use and the emergence of bacterial antimicrobial resistance (AMR) (1, 2). Recently, the World Health Organization (WHO) designated AMR a major threat to public health (3) and evidence is accumulating that resistant bacteria can be transmitted between humans, food producing animals, and companion animals (4–7). Antimicrobial stewardship (AMS) policies and practices aim to limit the development of AMR and improve patient outcomes by promoting the appropriate use of antimicrobial drugs (8, 9). Assessment of antimicrobial drug prescribing practice use is a key component of AMS, but antimicrobial drug use patterns in companion animals are less frequently reported than in food producing animals (10).

Practicing AMS is aided by the application of available clinical prescribing guidelines. Antimicrobial prescribing practices differ between countries in part due to national and transnational legislation such as in Scandinavia and the European Union aimed at promoting AMS (11, 12). Presently, there are no equivalent legislative restrictions on antimicrobial drug prescribing to small animals in the United States of America (13). Since 2011, the International Society for Companion Animal Infectious Disease (ISCAID) has published a series of guidelines on antimicrobial prescribing for urinary tract infection (UTI), companion animal respiratory disease and canine bacterial folliculitis to aid clinician decision-making (14–17). Recent reports suggest limited concordance with published guidelines by veterinary general practitioners (18, 19). In a study of prescribing patterns in 926 primary care practices, 44% of recurrent UTI and 67% of non-recurrent UTI were treated in accordance with the ISCAID guidelines, while only 22% of bronchitis episodes were treated per the guidelines (18).

The American Veterinary Medical Association Task Force on Antimicrobial Stewardship in Companion Animal Practice advocates periodic review of antimicrobial prescribing including the frequency of bacterial culture and susceptibility testing and the frequency of resistant and multidrug resistant (MDR) infections (20). The patterns of antimicrobial prescribing in veterinary emergency and critical care (ECC) settings have not been previously described. A study of human emergency room (ER) practice found that 39% of antimicrobial drug prescriptions were inappropriate (21). This observation raises questions regarding the appropriateness of prescribing in veterinary ECC settings. Small animal ECC practice presents unique challenges for antimicrobial stewardship, since ER cases are often seen on an outpatient basis with limited follow-up (22, 23). Moreover, small animals hospitalized in intensive care units are frequently infected by MDR pathogens. In one study, MDR bacteria comprised 27% of microbiological cultures from canine critical care patients, which likely hinders effective antimicrobial prescribing (24). In order to address the threat posed by AMR in small animal ECC practice, and to identify opportunities to improve AMS, the typical patterns of antimicrobial drug prescribing and the degree of adherence to published antimicrobial prescribing guidelines must first be established.

The present study therefore aimed to describe the antimicrobial prescribing patterns of the small animal ECC service of a North American veterinary teaching hospital. Specifically, the present study aimed to determine the proportion of ECC patients prescribed antimicrobials, to describe the antimicrobial classes used and for which conditions, and to use this information to evaluate adherence to published guidelines for management of respiratory disease and UTI (15, 17). Additionally, the present study aimed to determine the frequency of microbiological culture and susceptibility testing and the prevalence of MDR pathogens cultured from patients managed by the ECC service. It was hypothesized that small animal ER outpatients are more frequently prescribed antimicrobial drugs without antimicrobial culture than CC inpatients, that CC inpatients are more frequently prescribed multiple antimicrobial drugs than ER outpatients, and that patients with MDR bacterial infections have longer durations of hospitalization and higher case fatality rates than those without.

## Materials and Methods

### Data Collection

The electronic medical records of cats and dogs assessed at the Cornell University Hospital for Animals were prospectively queried every 2 weeks from January 1, 2017 to December 31, 2017 for all prescriptions of antimicrobial drugs (Supplementary Data 1). Search results were then manually curated by a single person (DLP) to include only those drugs prescribed by the ECC Service. Each prescription was then independently reviewed by two people (JM, SR) and the prescribing service adjudicated as either CC or ER. Patients temporarily hospitalized for diagnostic investigation or therapeutic management that were discharged within 24 h of their presentation were included in the ER group. Associated patient demographics (species, age, sex, reproductive status, bodyweight), outcome (discharge, death, euthanasia) and duration of hospitalization (in days) were recorded for each antimicrobial drug prescription. The final diagnoses were recorded for each case.

Within each hospital visit, separate prescriptions of distinct formulations of the same drug were combined. For example, intravenous and oral metronidazole prescribed to the same patient during the same visit were considered to be one prescription. In contrast, within a single hospital visit, prescriptions of drugs with distinct chemical composition were considered as separate prescriptions even if they shared class, type, indication, or spectrum of activity. For example, amoxicillin-clavulanate and ampicillin-sulbactam were listed separately. For each prescription, the antimicrobial drug, the drug class, and the total duration of therapy (in days) was recorded. The total number of distinct antimicrobial drugs prescribed for a single disease process was also recorded.

The medical record for each hospital visit associated with an antimicrobial drug prescription was reviewed and the condition for which the patient received antimicrobial drugs categorized as: collective disorders of the ears, eyes, nose, and throat (EENT), endocrine, gastrointestinal, musculoskeletal, neoplastic, neurological, reproductive, respiratory, sepsis, skin, tick-borne, or urinary (Supplementary Data 2). The number, site(s) and results of bacterial cultures (growth / no growth) and the susceptibility patterns of cultured organisms were recorded for each patient. Multidrug resistant pathogens were defined as those bacterial isolates that were non-susceptible to at least one drug in three or more antimicrobial categories, with the exception of antimicrobials to which the pathogen has inherent resistance, as previously described (25).

### Data Analysis

Following review of the medical records, each antimicrobial drug prescription was classified as either therapeutic or non-therapeutic as previously described (13). Briefly, non-therapeutic use included drugs provided prophylactically to patients undergoing chemotherapy or radiation therapy, drugs prescribed to patients without a documented infection, prescriptions of doxycycline for anti-inflammatory purposes and perioperative prophylaxis. Non-therapeutic prescriptions, those associated with incomplete medical records, repeat prescriptions performed by ECC personnel out-of-hours on behalf of other services for patients managed by other inpatient services were excluded from further analysis.

Each therapeutic prescription was then categorized as being based on confirmed infection, suspected infection or no evidence of infection (13). *Confirmed infection* was defined as disease with a positive bacterial culture, bacteria identified on fluid analysis, positive tick-borne disease PCR or serology testing with compatible clinical signs. *Suspected infection* included documentation of an open wound or purulent skin disease, presence of neutrophilic exudates without microscopically visible bacteria, surgical visualization of gastrointestinal perforation without bacterial culture, thoracic radiographs consistent with pneumonia without bacterial culture, purulent discharge from an orifice without bacterial culture. *No evidence of infection* was defined as disease without confirmed or suspected infection, and absent an alternate indication for antimicrobial drug use. This category included negative serologic titers and bacterial cultures without growth and if the word “preventative” was present in the medical record.

For patients with respiratory diseases and UTI, adherence to published guidelines was assessed using specific criteria (Table 1). For UTI, all of the respective criteria listed were required to be satisfied in order for the prescription to be judged *appropriate*. For example, a dog with stranguria and pollakiuria with *Escherichia coli* cultured from a urine sample collected by cystocentesis, that was treated for 7 days with twice daily amoxicillin-clavulanate would have been categorized as appropriately treated. Likewise, for respiratory tract infections, all of the respective criteria listed were required to be satisfied for the antimicrobial drug prescription to be deemed appropriate for the relevant condition. For example, a cat that received doxycycline for 7 days to treat lethargy, inappetence and a mucopurulent nasal discharge present for 10 days would have adjudicated to have received antimicrobials appropriately. If the duration of treatment exceeded that recommended in the relevant guidelines, the prescription was deemed inappropriate.

**Table 1:** Criteria to assess appropriateness of antimicrobial prescriptions.

**Urinary tract infection (simple)**	**Pneumonia in dogs and cats**
1. Urinalysis with sediment evaluation finding an active sediment 2. Urine culture performed a. Cystocentesis or bladder wall sample: CFU/mL > 10^3^ b. Male catheter: CFU/mL > 10^4^ c. Female catheter: CFU/mL > 10^5^ 3. Sample obtained by cystocentesis or catheterization 4. Therapy: a. Amoxicillin 11–15 mg/kg q8h *OR* b. Trimethoprim-Sulfamethoxazole 15 mg/kg q12h *OR* c. Amoxicillin-clavulanate 12.5–25 mg/kg q12h 5. Duration: 7–10 days	1. Cough, fever, lethargy, inappetence, tachypnea 2. Complete blood count 3. Thoracic radiographs supporting diagnosis of pneumonia 4. Lavage specimen for cytology and culture 5. Antimicrobials a. Mild pneumonia with no fever: Doxycycline b. Moderate clinical signs: 1st generation cephalosporin, amoxicillin, or amoxicillin clavulanate c. Suggestion of sepsis (hypoglycemia, injected mucous membranes): clindamycin or aminopenicillin, and fluoroquinolone d. Hypoxemia present: broad spectrum therapy (clindamycin or aminopenicillin, and fluoroquinolone or 3rd generation cephalosporin)* 6. Re-evaluation 10–14 days post starting therapy
**Upper respiratory tract disease in cats**	**Chronic infectious respiratory disease complex in dogs**
1. Presence of mucopurulent nasal discharge 2. Clinical signs >10 days or worsening of clinical signs after 5–7 days 3. Fever and/or lethargy and/or decrease in appetite 4. Doxycycline*, amoxicillin, or amoxicillin/clavulanate per clinical suspicion for absence of *Chlamydophila felis* or *Mycoplasma* or presence of beta-lactamase producing organism 5. Duration: 7–10 days	1. Acute onset cough with or without sneezing 2. Mucopurulent discharge for <10 days with fever and /or inappetence, and/or lethargy 3. Doxycycline 4. Duration: 7–10 days

Urinary tract infection criteria were adapted from Weese et al. (16). For simple urinary tract infections, all 5 criteria needed to be satisfied for therapy to be judged appropriate. Prescriptions were deemed inappropriate if they did not meet or exceed the recommended treatment time. Drug dosages were evaluated based on the drug table from the guidelines (15). Prescriptions were deemed appropriate if they were within 1 mg/kg of the guideline recommended dose. Prescriptions were deemed inappropriate if they were under dosed. Urine cultures were considered positive if the bacterial count was >10^3^ for cystocentesis samples and bladder wall samples, or >10^4^ for male catheterized samples and >10^5^ for female catheterized samples. Respiratory tract infection criteria were adapted from guidelines for treatment of respiratory tract diseases in dogs and cats (15). For respiratory diseases, all 4 (CIRD) or first 5 (pneumonia and URI) criteria needed to be satisfied for therapy to be judged appropriate. Asterisk (*) indicates a deviation from published guidelines based on clinical judgement.

### Statistical Analysis

Normality was assessed using the D'Agostino-Pearson test. Descriptive statistics were calculated for age, bodyweight, duration of hospitalization, and duration of antimicrobial prescription. Parametric data are summarized using mean ± SD, while non-parametric are summarized using median (min-max). Continuous variables were compared between groups using unpaired Student's *t*-test or the Mann-Whitney *U-*test. Relative frequencies in categorical data (infection status, culture acquisition, culture sites, survival, presence of MDR organisms on culture, drug class) were compared using Fisher's exact test or by Chi-square. *Post-hoc* Bonferroni corrections were applied to account for multiple comparisons. Alpha was set at 0.05. All analyses were conducted using commercial software (Prism 8.3, GraphPad, La Jolla, CA).

## Results

### Prescription Prevalence

**Figure 1:**
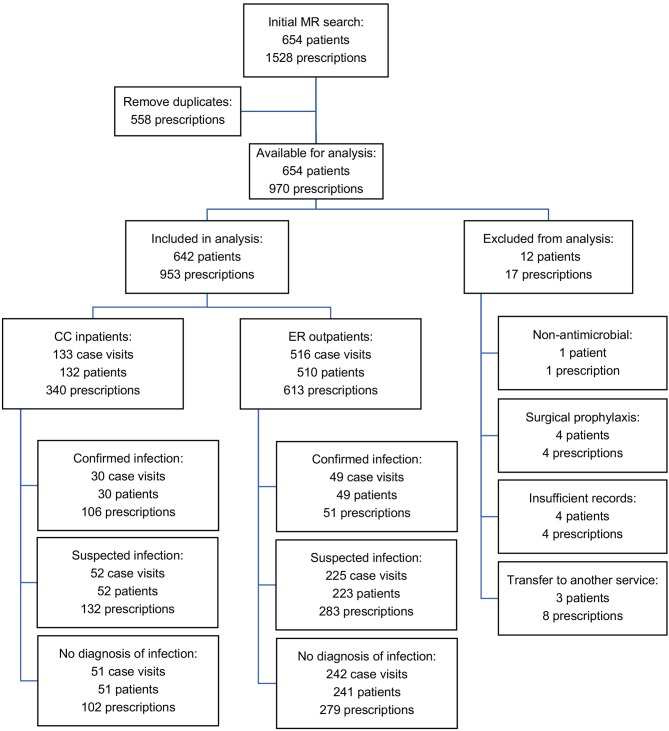
Flow chart of curation and analysis of medical records and prescriptions included in the final dataset.

A total of 1,528 antimicrobial drug prescriptions were identified, resulting from 654 patients over the 12-month study period. Eliminating duplicates (558 refills for hospitalized patients) left 970 prescriptions. Of these, 17 prescriptions from 12 patients were excluded from analysis (8 where cases were transferred to another service within the hospital, 4 cases received perioperative prophylaxis, 4 cases had incomplete medical records and 1 case had an antimicrobial prescribed for a non-antimicrobial indication). After curation, there were 953 prescriptions from 642 patients (517 dogs, 125 cats) available for analysis (Figure 1). Of these, 613 (64.3%) prescriptions were for ER outpatients and 340 (35.7%) were prescriptions for CC inpatients. Antimicrobial drugs were prescribed following 516 ER outpatient visits to 410 dogs and 100 cats. Six dogs had 2 ER visits each where they were prescribed antimicrobials. In 2017, there were 5,091 ER visits, of which 3,125 (61.4%) resulted in an outpatient event. Of these, 516 ER outpatient case visits resulted in 613 antimicrobial prescriptions to 510 animals, equivalent to an outpatient prescription rate of 16.5% (516/3,125). In 2017, the CC service managed 311 patients for 822 patient days. Antimicrobial drugs were prescribed during 133 CC inpatient case visits to 107 dogs and 25 cats. One dog had 2 separate CC inpatient episodes during which antimicrobial drugs were prescribed. Of these, 133 case visits (42.7%) resulted in 340 prescriptions, equivalent to an inpatient prescription rate of 42.8% (133/311). Descriptive statistics, duration of hospitalization, outcome and number of antimicrobials prescribed for the 642 patients included are summarized in Table 2.

**Table 2:** Summaries of patient demographics, duration of hospitalization, outcome and number of antimicrobials prescribed for ER and CC services.

	**Emergency room (outpatient)**		**Critical care (inpatient)**	
	**Dogs**	**Cats**	**Dogs**	**Cats**
No.	410	100	107	25
Sex (MI/MC/FI/FS)	65/137/41/167	8/53/9/30	9/47/13/38	4/14/0/7
Age (years)	6.01 (0.08–17.45)	6.14 (0.05–18.53)	6.17 (0.19–14.72)	6.61 (4.89)
Bodyweight (kg)	18.85 (1.00–102.00)	4.4 (1.598)	13.2 (1.30–61.0)	4.4 (1.513)
Duration of hospitalization (days)	N/A	N/A	2 (0–18)	3 (1–14)
Discharged	402 (96.6%)	98 (98.0%)	84 (77.8%)	16 (64%)
Euthanized	13 (3.1%)	2 (2.0%)	20 (18.5%)	6 (24%)
Died	1 (0.2%)	0 (0.0%)	4 (3.7%)	3 (12%)
No. of antimicrobial drugs prescribed				
1	343 (82.5%)	88 (88%)	29 (26.9%)	7 (28%)
2	64 (15.4%)	10 (10%)	24 (22.2%)	5 (20%)
3	8 (1.9%)	2 (2%)	35 (32.4%)	5 (20%)
4	1 (0.2%)	0 (0%)	12 (11.1%)	7 (28%)
5	0 (0.0%)	0 (0%)	5 (4.6%)	0 (0%)
6	0 (0.0%)	0 (0%)	2 (1.9%)	1 (4%)
7	0 (0.0%)	0 (0%)	1 (0.9%)	0 (0%)

*The mean and standard deviation are displayed for normally distributed data and the median and min–max are displayed for non-normally distributed data. The total number of patients seen is not equal to the sum of patients discharged, euthanized and died because some patients were seen and prescribed antimicrobials more than once. The number of patients therefore represents the total individuals treated, rather than the total number of visits. MI, male intact; MC, male castrated; FI, female intact; FS, female spayed; No., number*.

### Prescription Indications

**Figure 2:**
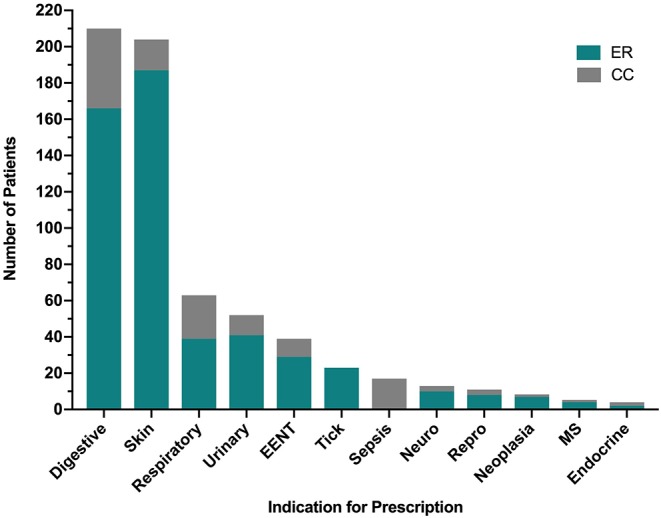
Indications for antimicrobial prescription by category from the Emergency Room (ER) and Critical Care (CC) services. Specific diagnoses included in each of the 12 categories are detailed in Supplementary Data 2. EENT, disorders of the ears, eyes, nose and throat; MS, musculoskeletal.

The most common indications for antimicrobial drug prescription for ER outpatients included conditions of the skin (227 prescriptions, 37.0%) from 187 outpatient visits, gastrointestinal disorders (173 prescriptions, 28.2%) from 166 outpatient visits, respiratory disease (50 prescriptions, 8.2%) from 39 outpatient visits, urinary tract disease (44 prescriptions, 7.2%) from 41 outpatient visits, EENT disorders (34 prescriptions, 5.5%) from 29 outpatient visits and tick-borne disease (24 prescriptions, 3.9%) from 23 outpatient visits (Figure 2). The most common indications for antimicrobial drug prescription for CC inpatients included gastrointestinal disorders (106 prescriptions, 31.2%) from 44 inpatient visits, respiratory disease (71 prescriptions, 20.9%) from 24 inpatient visits, sepsis (61 prescriptions, 17.9%) from 17 inpatient visits, conditions of the skin (41 prescriptions, 12.1%) from 17 inpatient visits, urinary tract disease (26 prescriptions, 7.6%) from 11 inpatient visits and EENT disorders (23 prescriptions, 6.8%) from 10 inpatient visits (Figure 2). Neurologic disease, reproductive disease, neoplasia, musculoskeletal disease, and endocrine related diseases each represented <2.5% of case visits for ER and CC. No cases of tick-borne disease were treated by the CC service and no cases of sepsis were treated by the ER service. Summary data for the most common disease categories are presented in Table 3.

**Table 3:** Most frequently prescribed drugs (top 5) and duration for skin, respiratory, and gastrointestinal disease prescribed by the emergency room and critical care services.

**Disease category** **total number of Rx**	**Drug**	**Number of prescriptions** ***N* (%)**	**Median duration** **days (range)**	**Drug**	**Number of prescriptions** ***N* (%)**	**Median duration** **days (range)**
	Emergency room			Critical care		
Skin ER 227 Rx CC 41 Rx	Amoxicillin/Clavulanate	152 (67.0%)	7 (2.5–17)	Ampicillin/Sulbactam	13 (31.7%)	2 (1–7)
	Ampicillin/Sulbactam	31 (13.7%)	1 (1)	Amoxicillin/Clavulanate	8 (19.5%)	10.1 (3.0)
	Cephalexin	25 (11.0%)	10 (3.5–42)	Enrofloxacin	5 (12.2%)	12 (2–16)
	Enrofloxacin	6 (2.6%)	7 (1–28)	Cephalexin	3 (7.3%)	5 (5–10)
	Cefovecin	5 (2.0%)	N/A	Metronidazole	2 (4.8%)	6 (2–10)
Respiratory ER 50 Rx CC 71 Rx	Amoxicillin/Clavulanate	15 (30.0%)	9.3 (3.3)	Ampicillin/Sulbactam	22 (31.0%)	3.7 (1.9)
	Doxycycline	14 (28.0%)	10 (7–15.5)	Enrofloxacin	21 (29.6%)	9.6 (5.8)
	Enrofloxacin	11 (22.0%)	6.5 (5.9)	Amoxicillin/Clavulanate	14 (19.7%)	10.9 (4.1)
	Ampicillin/Sulbactam	4 (8.0%)	1 (1)	Ceftazidime	3 (4.2%)	3 (2–5)
	Ampicillin	1 (2%)	1	Metronidazole	3 (4.2%)	5 (1–11)
	Azithromycin	1 (2%)	4			
	Clindamycin	1 (2%)	1			
	Metronidazole	1 (2%)	10			
Gastrointestinal ER 173 Rx CC 106 Rx	Metronidazole	130 (75.1%)	5.5 (1–15)	Metronidazole	40 (37.3%)	6 (1–18)
	Amoxicillin/Clavulanate	12 (6.9%)	7 (7,–14)	Ampicillin/Sulbactam	27 (25.4%)	2.6 (1.4)
	Cefovecin	11 (6.3%)	5.8 (4.7)	Enrofloxacin	17 (16.0%)	7.6 (7.2)
	Enrofloxacin	9 (5.2%)	NA	Amoxicillin/Clavulanate	11 (10.3%)	9.5 (4.3)
	Ampicillin/Sulbactam	7 (4.0%)	1 (1,2)	Cefazolin	3 (2.8%)	1 (1)

*Rx, prescriptions; NA, not available. The mean and standard deviation are displayed for parametric data and the median and minimum and maximum values are displayed for non-parametric data. The total number of prescriptions from the table does not equate to the sum of antimicrobials within the table as more than 5 drugs were prescribed for those disease categories*.

### Characterization of Antimicrobial Drug Prescriptions

**Figure 3:**
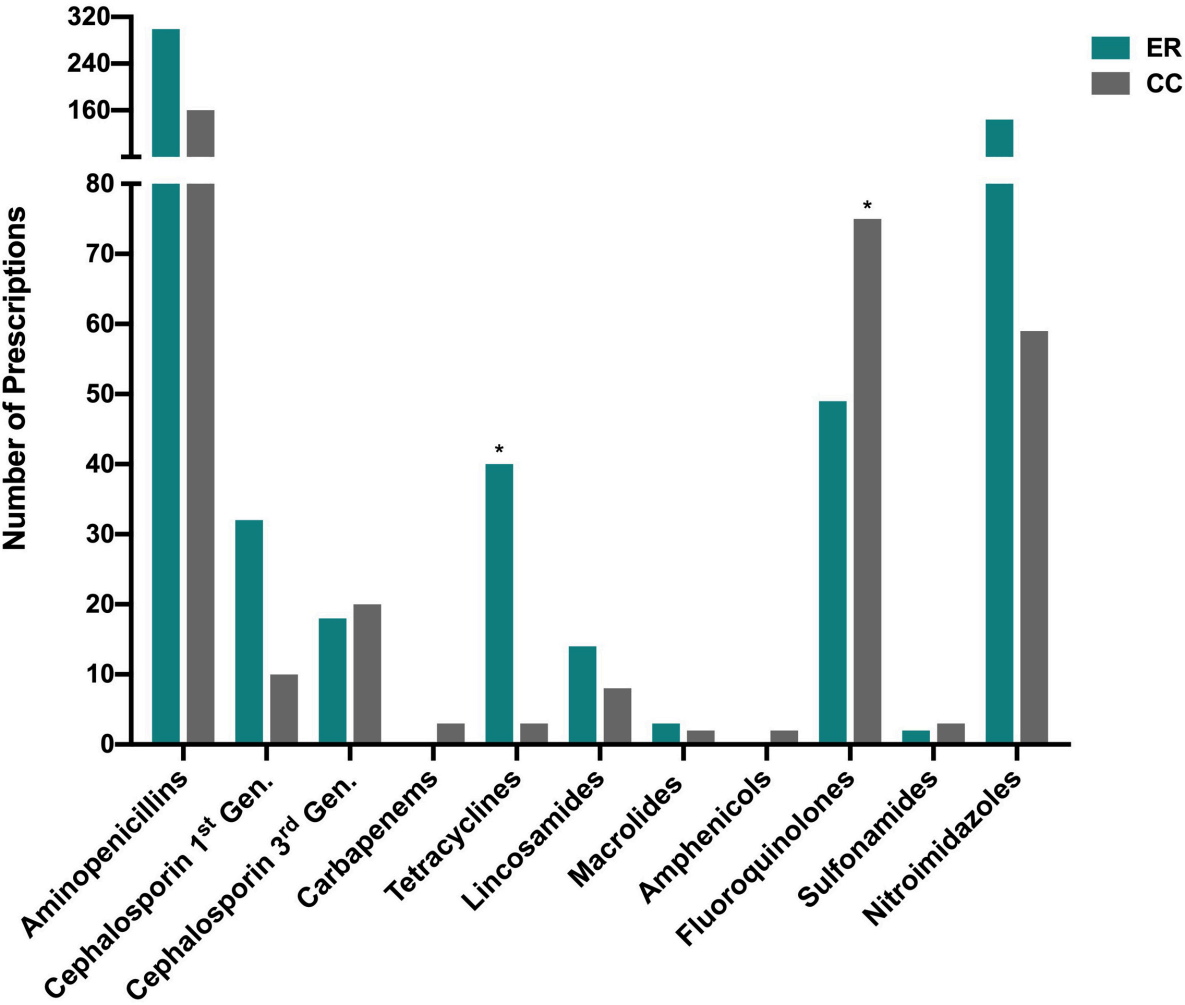
Distribution of drug classes prescribed by the Emergency Room (ER) and Critical Care (CC) services. The (*) symbol denotes that a significant difference (after correction for multiple comparisons) existed in the prescribing frequency of the labeled drug class between ER outpatients and CC inpatients (*P* < 0.0001). All classes prescribed are represented in the figure, i.e., drug classes for which zero prescriptions were recorded were omitted. Gen, Generation.

The antimicrobial drug classes most frequently prescribed were aminopenicillins (459/953, 48.2%), nitroimidazoles (203/953, 21.3%), and fluoroquinolones (124/953, 13.0%). All other drug classes each represented <5% of total prescriptions. Third generation cephalosporins represented 4.0% (38/953) of all prescriptions (Figure 3). The most frequently prescribed drugs were amoxicillin-clavulanate (294/953, 30.4%), metronidazole (205/953, 21.5%), ampicillin-sulbactam (158/953, 16.6%), enrofloxacin (123/953, 12.9%), doxycycline (43/953, 4.5%), cephalexin (33/953 3.5%), and clindamycin (22/953 2.3%). The most commonly prescribed drugs for the ER were amoxicillin/clavulanate (243/613, 39.6%), metronidazole (146/613, 23.8%), and ampicillin/sulbactam (55/613, 9.0%). The most commonly prescribed drugs for the CC service were ampicillin/sulbactam (103/340, 30.3%), enrofloxacin (75/340, 22.1%), and metronidazole (59/340, 17.4%). All other antimicrobial drugs each represented <2% each of total prescriptions. Fluoroquinolones were more frequently prescribed by the CC service (21%) compared to ER (8%) *P* < 0.0001. Tetracyclines were more frequently prescribed by the ER (6%) compared to CC (0.8%) *P* < 0.0001. All prescriptions for chloramphenicol (*n* = 2), meropenem (*n* = 2), and imipenem (*n* = 1), were for patients managed by the CC service and were based on bacterial susceptibility data. As described in Table 2, antimicrobial polypharmacy was common in both services. A significantly larger proportion of CC inpatients were prescribed >2 drugs as compared to ER outpatients (*P* < 0.0001).

For both ER and CC, the duration of antimicrobial administration was calculated to include in-hospital administration and the duration of antimicrobials prescribed for continued therapy after hospital discharge. Patients were most frequently prescribed between 1 and 7 days of antimicrobial drugs (66.4% overall, 68.5% CC, 65.2% ER), followed by 8–14 days (25.7% overall, 23.9% CC, 26.8% ER) and 15–28 days (6.2% overall, 7.3% CC, 5.6% ER). Overall, 1.6% patients (0.3% CC, 2.4% ER) received antimicrobial drugs for 29–42 days, most of which were treated for tick-borne disease. Patients with sepsis were treated for a median of 11 days (1-34). There were insufficient records to determine the length of antimicrobial drug prescription in 26 prescriptions, 24 of which were for topical ocular medications or for topical dermatologic or otic preparations. Topical medications included in analysis comprised: ophthalmic medications (bacitracin-neomycin-polymyxin ointment, neomycin-polymyxin-dexamethasone ointment, cefazolin ointment, ofloxacin ointment, erythromycin ointment), and otic medications (gentamicin sulfate-mometasone furoate monohydrate-clotrimazole ointment, gentamicin sulfate-betamethasone valerate-clotrimazole ointment, miconazole nitrate-polymyxin B sulfate-prednisolone acetate suspension and thiabendazole-dexamethasone-neomycin sulfate solution). Cefovecin was prescribed for 17 ER outpatient visits, all of which received a single injection. The majority (11/17, 64.7%) were for gastrointestinal disorders.

### Bacterial Culture and Susceptibility Testing

**Figure 4:**
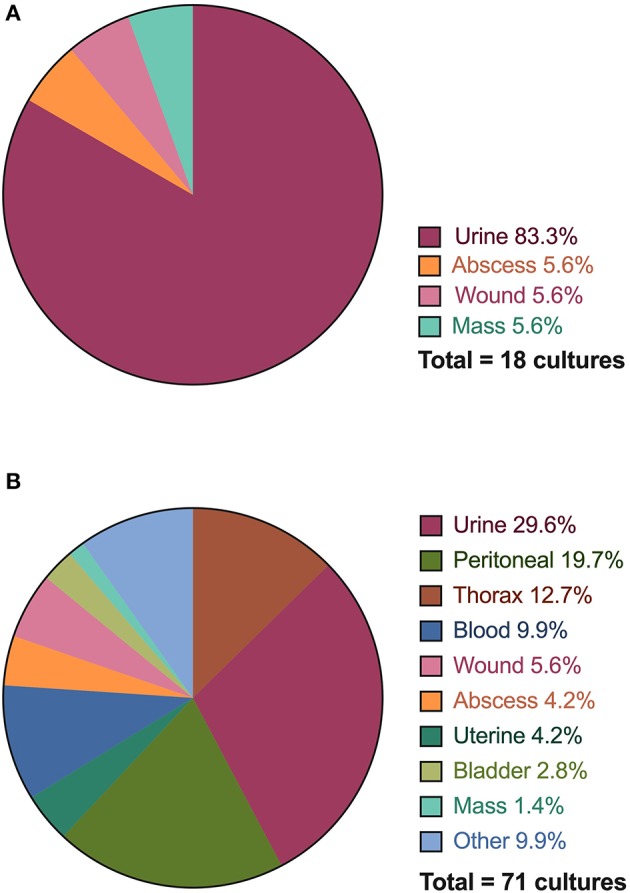
Distribution of anatomical sites and specimens collected for bacterial culture and susceptibility testing performed for Emergency Room (ER) outpatients **(A)** and Critical Care (CC) inpatients **(B)**. The thorax category includes both pleural cavity and lung aspirate samples.

From the 649 case visits (133 CC, 516 ER) for which antimicrobial drugs were prescribed, 89 samples from various anatomic sites were submitted for bacterial cultures (Figure 4). Of these, 71/89 (79.7%) were collected from CC inpatients, and 18/89 (20.2%) collected from ER outpatients (Table 4). For ER outpatients, this represents a sample submission rate of 3.5% (18/516), while for CC inpatients, this represents a sample submission rate of 34.6% (46/133). All ER outpatients had 1 culture sample submitted while 30/46 (65.2%) CC patients had 1 sample submitted, 10/46 (21.7%) had 2 samples submitted, 4/46 (8.7%) had 3 samples submitted, and 4 and 5 samples were submitted for one patient each (1/46, 2.2% each). All ER outpatients had only a single anatomic site cultured while CC patients had up to 3 distinct sites cultured per patient, typically patients with sepsis or respiratory disease.

**Table 4:** Culture and susceptibility results from 649 cases prescribed antimicrobials by the emergency and critical care service in 2017.

	**All cases**	**Emergency room**	**Critical care**
Number of patients with cultures performed	64	18	46
Number of cultures performed	89	18	71
Number of positive cultures	35	9	26
Number of pathogens isolated	63	8	55
Number of MDR pathogens	14	2	12
Number of cases with MDR pathogen (number of pathogens)			
Sepsis	5 (8)	0	5 (8)
Urinary	2 (2)	1 (1)	1 (1)
Skin	1 (1)	1 (1)	0
Neurologic	1 (1)	0	1 (1)
Digestive	2 (2)	0	2 (2)

*MDR (multi-drug resistant) pathogens were defined as bacterial isolates which were found via a susceptibility panel to be non-susceptible to at least one agent in three or more antimicrobial categories, with the exception of antimicrobials to which the pathogen has inherent resistance, as previously described (25)*.

For all infections, 54/64 (84.4%) cultures were performed on the same site as the system identified as the indication for prescription. Urine cultures represented the majority of cultures submitted through the emergency room (15/18, 83.3%), followed by abscesses (1/18, 5.6%), wounds (1/18, 5.6%), and masses (1/18, 5.6%). The critical care service performed cultures on urine (21/71, 29.6%), peritoneal effusion (14/71, 19.7%), thorax (pleural effusion, lung aspirate) (9/71, 12.7%), blood (7/71, 9.9%), and wounds (4/71, 5.6%). All other locations represented <5% of cultures. Of the 89 cultures performed, 35/89 (39.3%) had growth yielding 63 pathogens. Sixteen fastidious or anaerobic pathogens did not have a susceptibility reported. The largest number of bacterial isolates were identified in patients with sepsis (41.2% of pathogens), urinary disorders (20.6% of pathogens), and skin disease (15.9%) followed by respiratory (14.3%) reproductive (6.3%), neurological diseases (4.8%), digestive (3.2%), and endocrine (1.6%). No cultures were submitted for patients diagnosed with musculoskeletal disease, neoplasia or tick-borne disease. Of the 23 patients prescribed antimicrobial drugs for tick-borne disease, 17 had a positive test result for *Borrelia* or *Anaplasma* exposure using a point-of-care immunochromatographic test (SNAP 4DX, IDEXX, Westbrook, ME).

For ER, 2 of 8 (25%) bacterial isolates grown on culture were MDR. For CC 12/55 (21.8%) of bacterial isolates were MDR. There was no difference in the frequency of MDR isolates between services (*P* > 0.999). There was no difference between the frequency of ER outpatients with a positive bacterial culture (9/18, 50%) compared to CC inpatients (26/46, 56.5%), *P* = 0.781. Patients with sepsis had the largest proportion of patients (5/11, 45.4%) in which MDR infections were identified. There was no difference in the frequency of survival to discharge of CC inpatients infected with an MDR isolate (6/9) compared to those without MDR isolates (12/17), *P* > 0.999. The median length of hospitalization for CC patients with an MDR isolate identified was 9 days (3-14) and for those without an MDR isolate identified was 5 days (1-18). The hospital length of stay values were not significantly different between these two groups (*P* = 0.25).

**Figure 5:**
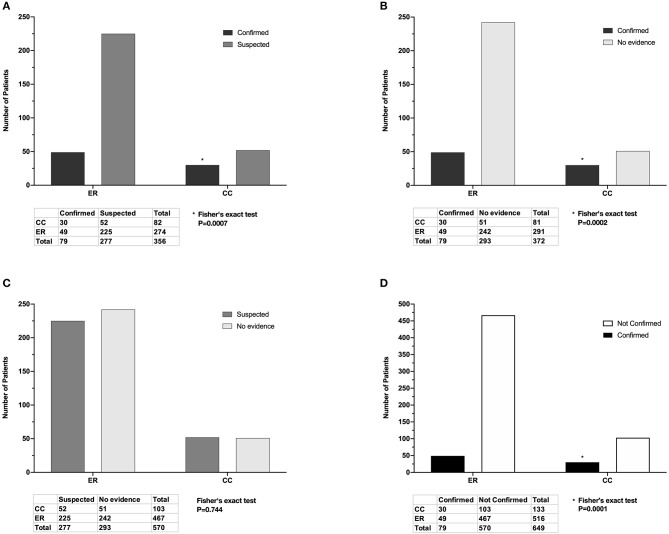
Adjudicated infection status of cases seen by the Emergency Room (ER) and Critical Care (CC) services and prescribed antimicrobial drugs. **(A)** There was a significantly higher proportion of confirmed infections than suspected infections in CC inpatients compared to ER outpatients. **(B)** There was a significantly higher proportion of confirmed infections than those with no evidence in CC inpatients compared to ER outpatients. **(C)** There was no significant difference in the proportions of suspected infection and no evidence between ER outpatients and CC inpatients. **(D)** There was a significantly higher proportion of confirmed infections compared to unconfirmed infections (suspected plus no evidence) in CC inpatients compared to ER inpatients. Listed *P*-values for **(A,B,D)** marked with (*) were significant after Bonferroni correction for multiple comparisons.

In total, 79/649 (12.2%) cases prescribed antimicrobial drugs met criteria for confirmed infection, 277/649 (42.7%) met criteria for suspected infection, and 293/649 (45.1%) had no evidence of infection (Figure 1). A significantly higher proportion of CC inpatients had confirmed infection (30/133, 22.6%) compared to ER outpatients (49/516, 9.5%) *P* = 0.0001 (Figure 5).

### Adherence to Prescribing Guidelines

Overall, 52 patients were prescribed antimicrobials for disorders of the urinary tract including simple UTI (*n* = 38), acute kidney injury (*n* = 7), pyelonephritis (*n* = 3), chronic kidney disease (*n* = 2), uroperitoneum (*n* = 1), and prostatitis (*n* = 1). The antimicrobial prescriptions for 13/38 (34%) patients with simple UTIs were classified as appropriate (Table 5). The most frequently unsatisfied criteria were failure to submit a urine sample for culture, which was performed in only 45% of patients treated for a UTI and assessment and/or submission of a non-catheter or cystocentesis sample, which occurred in 37% cases.

**Table 5:** Summary of assessments of the degree to which antimicrobial prescriptions for urinary tract and for respiratory tract infections were appropriate based criteria detailed in Table 1.

**Urinary tract infection**	***N* (%)**
Total	38
Urinalysis performed with active sediment present	33 (87%)
Catheter or cystocentesis sample	24 (63%)
Urine culture submitted	17 (45%)
°*Positive growth with appropriate CFU/mL*	*9 (24%)*
Drug choice	30 (79%)
Duration	31 (81%)
Full compliance	13 (34%)
**Pneumonia**	***N*** **(%)**
Total	28
Presence of clinical signs	28 (100%)
Complete blood count	22 (78%)
Thoracic radiographs with evidence of pneumonia	28 (100%)
Culture and susceptibility on respiratory tree sample	2 (7%)
Drug choice and dosage based on perceived illness severity:	21 (75%)
Moderate disease with beta-lactam	2
Evidence of sepsis	8
Hypoxemia with combination therapy	11
Full compliance	2 (7%)
**Canine infectious respiratory disease complex**	***N*** **(%)**
Total	11
Acute cough	11 (100%)
Mucopurulent discharge with either fever and/or lethargy and/or inappetence	1 (9%)
Doxycycline	10 (90%)
Duration 7–10 days	8 (73%)
Full compliance	1 (9%)
**Upper respiratory infection**	***N*** **(%)**
Total	8
Presence of mucopurulent nasal discharge	4 (50%)
Clinical signs <10 days or worsening after 5 to 7 days	11 (100%)
Fever and/or lethargy and/or inappetence	4 (50%)
Doxycycline	0 (0%)
Duration 7–10 days	6 (75%)
Full compliance	2 (25%)^*^

* *2 cats were prescribed topical erythromycin ointment and no systemic antimicrobials*.

A total of 62 patients were prescribed antimicrobial drugs for respiratory disease including pneumonia (*n* = 28), canine respiratory disease complex (*n* = 11), feline upper respiratory tract infection (*n* = 8), pyothorax (*n* = 2), feline asthma, pyogranulomatous laryngitis, neoplasia, and non-cardiogenic pulmonary edema (all *n* = 1). A final diagnosis was not established for 9 cases. One patient was seen on two separate occasions. The antimicrobial prescriptions for 2/28 (7%) cases with pneumonia were classified as appropriate (Table 5). This was typically due to lack of sample collection for culture and susceptibility testing. Additional criteria that were unsatisfied included lack of a complete blood count or smear review and incorrect drug selection based on clinical severity. Two patients received under-dosed medications. Of the 11 dogs diagnosed with canine infectious respiratory disease complex (CIRDC), only 1 met all the criteria for appropriate prescription since most cases did not have a mucopurulent nasal discharge in association with fever, lethargy and or inappetence. Similarly, only 2/8 cats with respiratory infections were appropriately treated, typically due to incorrect drug selection.

## Discussion

The present study focuses on antimicrobial prescriptions in a small animal ECC setting and aimed to evaluate prescribing frequency, patterns, and practices and to assess compliance with published antimicrobial stewardship guidelines. The antimicrobial prescription rate was 16.5% in our ER outpatient population. This is comparable with data from a recent survey of UK general practices that found antimicrobials were the second most commonly prescribed drugs (after vaccines) and comprised 17.6% of canine prescriptions and 14.5% of feline prescriptions (26). To the authors' knowledge, the frequency of antimicrobial drug prescription for companion animal ER outpatients has not been previously reported. The rate of antimicrobial prescribing to ER outpatients in the present study was similar to that in human medical patient populations, where rates of 13.6% for adult ER (27) and 19.9% for pediatric ER visits (28) are reported. Although the overall rate of prescribing is comparable with human ER populations, it is likely that this still represents overprescribing. In the present study, 45.1% of all cases prescribed antimicrobial drugs had no evidence of infection, consistent with a previous study (13). Since our definitions of confirmed and suspected infection were broad and potentially included patients that were only at-risk of an infection (e.g., open wounds) the true rate of overprescribing may be higher. This finding is of concern, but the rate of inappropriate antimicrobial drug prescribing is similar that reported for human ER outpatient populations, where rates of 39–52% are reported (21, 29).

In the present study, the aminopenicillins, nitroimidazoles, and fluoroquinolones were the most frequently prescribed antimicrobial drug classes. This is similar to that reported for a comparable US veterinary teaching hospital (13). Consistent with these drug classes, the most frequently prescribed individual drugs were amoxicillin-clavulanate (30.4%), metronidazole (21.5%), ampicillin-sulbactam (16.6%), and enrofloxacin (12.9%). Fluoroquinolones have been extensively prescribed in veterinary medicine, and there has been a contemporaneous increase in bacterial fluoroquinolone resistance worldwide in both humans and companion animals. Indeed, recent studies have suggested that resistance mechanisms for fluoroquinolones are the same in companion animals and humans (30). The potential for AMR development in bacteria with zoonotic potential and in potential opportunistic pathogens led to limitation of the use of fluoroquinolones in food producing animals in some countries (31). In addition, several Scandinavian and European countries have significantly restricted the use of fluoroquinolones in all veterinary species, including companion animals (32) and a subsequent decrease in fluoroquinolone resistance in these countries has been documented (33). In the present study, fluoroquinolones were more frequently prescribed by the CC service compared to the ER. This may reflect a greater illness severity (perceived or existent) in CC patients, or a greater incidence of patients with sepsis or infections by Enterobacteriaceae in CC compared to ER. The fluoroquinolones are often prescribed to increase coverage against gram-negative organisms and may be less nephrotoxic than aminoglycosides. Fluoroquinolone prescribing in the present study represents a clear opportunity to improve antimicrobial stewardship through better implementation of antimicrobial de-escalation strategies.

In contrast, the present study suggests infrequent usage of third generation cephalosporins compared to the fluoroquinolones. Third generation cephalosporins are also considered by the WHO to be critical for human health, and similarly to fluoroquinolones are targets of European antimicrobial stewardship programs that aim to decrease usage (34). Cefovecin is a widely used drug for cats in the UK (35, 36) and is also commonly prescribed in Switzerland (37). Ease of administration and excellent owner compliance are the most commonly cited reasons for administration of cefovecin (37, 38). In the present study, most ER cefovecin prescription were for outpatient treatment of parvovirus infections in dogs per the protocol described by Venn and others (39). Evaluation of a similar outpatient protocol using an antimicrobial other than cefovecin may be warranted from an antimicrobial stewardship perspective.

In the present study, 34% of UTIs were treated appropriately per the 2011 antimicrobial use guidelines. Using a different 10-point metric to gauge rationale for antimicrobial prescribing in a human ER ~25.4% of drug prescriptions were inappropriate based on indication, which is the closest surrogate to the criteria used in the present study (21). Although urine was the most frequently submitted sample type in the present study, failure to obtain a urine culture remained the primary cause of a lack of adherence to veterinary prescribing guidelines for UTI. We speculate that the nature of emergency practice, as distinct from primary care, limited the ability of ER clinicians to readily obtain, submit and follow-up on urine cultures. The 2011 guidelines recommended urine sediment analysis be performed by trained personnel in a central laboratory. Outside normal business hours, access to central laboratories is limited which precludes obtaining results in a timely manner. Alternatives to culture for ER outpatients might include modified Wright staining or Gram staining of urine sediment to enhance detection of bacteriuria in dogs (40–42). Given the findings of the present study, incorporating additional urine sediment staining into standard operating procedures for urinalysis might be beneficial as part of an antimicrobial stewardship program. Pollakiuria associated with UTI may render the bladder small, precluding ease of sampling since performing cystocentesis on a small urinary bladder may not be feasible or safe, while urinary catherization commonly requires sedation and is infrequently performed for diagnostic purposes. There may also be financial constraints that limit culture submission. In a recent survey of veterinarians from the north western United States cost of culture and sensitivity was a commonly listed barrier (43). The antimicrobial use guidelines for UTI were recently updated (17) and incorporate a change in the recommended duration of antimicrobial therapy to 3–5 days rather than 7–10 days previously suggested. The 2011 guidelines were current during the time of the present study. Interestingly, only 1 prescription in the present study would have complied with the newly recommended duration of therapy. It should be noted, however, that the optimal duration of antimicrobial therapy is currently unknown for most diseases in veterinary medicine.

Similar to the situation for UTI, a large proportion of patients in the present study with respiratory disease that received antimicrobial drugs did not meet published criteria for therapy. For instance, 75% of cats treated for upper respiratory infections and 91% of dogs treated for CIRDC did not warrant antimicrobial therapy per the current guidelines (15). It should be noted, however that the guidelines were published in March of 2017—the year that data collection for the present study was undertaken. As such, it is unlikely that the guidelines had a substantial impact on contemporaneous prescribing behavior. As for UTI, the primary reason for non-adherence to published guidelines was the lack of culture and susceptibility testing from the respiratory system. Although various techniques are described to obtain respiratory tree samples, these procedures can be challenging and risky to perform in patients with respiratory distress or hypoxemia. Unfortunately, straightforward alternative approaches (e.g., deep oral swabs) are not adequate substitutes (44). The 2017 guidelines recommend prescribing broad spectrum antimicrobial drugs to dogs and cats with pneumonia if they exhibit signs of sepsis, such as injected mucous membranes or hypoglycemia. This is problematic because it is well recognized that sepsis manifests variably and is very challenging to define in both veterinary and human medicine (45–47). As such, the 2017 guidelines may be difficult to apply clinically since dogs and cats might have bacteremia from pneumonia without injected mucous membranes or hypoglycemia (48). We speculate that a criterion of “requirement for oxygen supplementation” might be an easier and more inclusive benchmark for antimicrobial selection in pneumonia. Hypoxemia suggests underlying organ dysfunction and would in our opinion warrant broad spectrum antimicrobial administration to suspected pneumonia cases. Indeed, using this criterion to adjudicate prescribing in the present study suggests that drug selections were appropriate in 75% of cases.

Most dogs with pneumonia were prescribed antimicrobial drugs for 8–14 days. The 2017 guidelines suggest patient reassessment 10–14 days after initiation of therapy because not all patients will require 4–6 weeks of therapy. One limitation of assessing the adequacy of prescribing to ER outpatients in the present study is the lack of follow-up. Prospective studies evaluating the efficacy of different durations of antimicrobials for pneumonia are urgently required to better guide small animal clinicians. The use of biomarkers including C-reactive protein might aid in this adjudication since this has been shown to significantly decrease duration of antimicrobials in both humans (49) and dogs (50) with pneumonia without negatively affecting outcome. Similarly, patients suffering from sepsis were treated for a median of 11 days (1-34). In humans, survival rates are not different in patients with sepsis due to complicated intra-abdominal infection who receive short vs. long courses of antimicrobials (51). In addition, duration of antimicrobial drug therapy is positively associated with risk of subsequent extra-abdominal infection and mortality (52). Current human recommendations for abdominal infections with adequate source control are therefore to limit antimicrobial drugs to 7 days or fewer (53, 54). Comparing the results of the present study to the human guidelines suggests that it may be feasible and safe to reduce the duration of antimicrobial drug administration in small animals also.

Gastrointestinal disease was a frequent indication for antimicrobial prescribing in the present study and metronidazole was the second and third most frequently prescribed drug to ER and CC patients, respectively. Acute diarrhea, including acute hemorrhagic diarrhea syndrome is frequently encountered in dogs and cats. This condition is commonly treated with metronidazole, but several recent publications suggest that the disease is typically self-limiting and that probiotic administration can result in as rapid a resolution of clinical signs as antimicrobial therapy (55, 56). It has been argued that in the absence of sepsis, antimicrobial therapy is not justified for management of acute diarrhea in small animals (57).

It is noteworthy that a significantly larger proportion of CC inpatients vs. ER outpatients had *confirmed infection* (22.6 vs. 9.5%). This may be due to differences in the level of training of CC vs. ER personnel, a perceived higher frequency of AMR in CC inpatients, greater ease of obtaining samples for bacterial culture from hospitalized inpatients and preselection of a client population with more extensive financial resources that facilitate diagnostic testing. In the present study, there was no significant difference between the proportion of positive bacterial cultures from ER outpatients compared to CC inpatients. Likewise, the proportion of MDR organisms identified was not significantly different, although the odds ratio was 1.94 (95% CI 0.22–5.97). This may be due to the small sample size (total *n* = 35) and larger study with the same proportions might suggest that CC patients that have prolonged durations of hospitalization have an increased likelihood of resistant infection as has been previously reported (24, 58). Since culture submission was not universally performed and therefore relied on clinician discretion and client financial means it is possible that only the most severely or chronically affected ER outpatients had cultures submitted and hence diminished the apparent difference between ER and CC patients.

Multidrug resistant infections were most frequently identified in patients with sepsis. There was no significant difference in the length of stay or the outcome of CC inpatients with MDR infections vs. those without, but this may be due to the small sample size since only 9 CC inpatients had MDR infections. Given the large retrospective nature of this study, we did not seek to identify possible causes for MDR bacteria in the CC or ER patients. It is likely that these patients had received previous antimicrobial administration and or had prolonged hospital stay prior to the culture sample being obtained (24, 58). Carbapenems and chloramphenicol were occasionally prescribed to animals with sepsis and MDR infections based on susceptibility results. This follows the ISCAID guidelines for use of carbapenems which recommends treatment where culture and susceptibility results suggest carbapenem susceptibility and resistance to reasonable alternatives, a treatable infection and only after consultation with an infectious disease expert. A recent retrospective study describing the usage of carbapenem in a similar institution demonstrated a similar low prescription rate. However, de-escalation to a lower tier antimicrobial or no antimicrobial was rarely performed in that study (59). The WHO has classified carbapenems on the “Watch” group of antimicrobials (60), which overlaps with the highest priority agents on the list of critically important antimicrobial drugs for human medicine. It is interesting to note that use of carbapenems is prohibited in any veterinary species in some European countries.

The present study has some limitations. The retrospective design precluded precise determination of the rationale for antimicrobial drug prescribing, particularly when more than one disease entity coexisted (e.g., pneumonia and diarrhea). It was not possible to fully evaluate adherence to prescription guidelines per ISCAID because our medical records searches were conducted by identifying antimicrobials prescriptions. As such, it was not possible to determine how many patients with clinical signs of a UTI or respiratory disease were not prescribed antimicrobials in accordance with guideline recommendations. Some patients may also have received antimicrobials prior to their ER visit which would likely have altered clinician decision-making. It was not feasible to retrospectively review the prior medical histories and records supplied by referring veterinarians that might have provided this information. In addition, we were unable to follow ER outpatients after discharge to determine when antimicrobials were discontinued or whether additional medications were subsequently prescribed by primary care veterinarians. The relatively small number of patients for which cultures were submitted also limited comparisons between ER and CC services.

In summary, the present study offers insights into current prescription practices of the ECC service of a large tertiary referral teaching hospital. Overall prescribing practices were consistent with other veterinary patient populations and compliance with published guidelines was comparable with the situation in human medicine. There were likely a substantial number of cases that were prescribed antimicrobials unnecessarily, however and closer adherence to published guidelines would be desirable. Education and antimicrobial stewardship programs have been effective in human emergency room in decreasing the number of inappropriate prescriptions (61), and implementation of a targeted antimicrobial stewardship training program and in house guidance might be of value in veterinary ECC settings.

## Data Availability Statement

Datasets used for this study are available upon reasonable request from the authors.

## Ethics Statement

Ethical approval for this study was not required from local institutional ethics committee approval because it presents a retrospective analysis of antimicrobial prescription as part of clinician-driven care provided to patients at the institution hospital. No client or patient identifying information is presented.

## Author Contributions

SR curated and analyzed data and co-wrote the manuscript. RG assisted with study design, collected and analyzed data, and co-wrote the manuscript. DL-P collected and curated the data and edited the manuscript. GL contributed to study design, data curation, and edited the manuscript. JM designed the study, assisted with data curation and analysis, and co-wrote the manuscript.

### Conflict of Interest

The authors declare that the research was conducted in the absence of any commercial or financial relationships that could be construed as a potential conflict of interest.
