# Supplementary material for: Antimicrobial Prescribing Practices in Small Animal Emergency and Critical Care
Source: Front Vet Sci. 2020 Feb 28;7:110. doi: 10.3389/fvets.2020.00110 (PMC7093014; doi:10.3389/fvets.2020.00110)
Supplement: Supplementary file 1 [file Data_Sheet_1.docx]

**Supplementary Data**

Supplementary Data 1. Antimicrobial drugs included in medical record searches

| Antimicrobial Drugs Included in Medical Records Search | |
| --- | --- |
| Amikacin Sulfate | Linezolid |
| Amoxicillin | Marbofloxacin |
| Amoxicillin/Clavulanate | Meropenem |
| Ampicillin | Metronidazole |
| Ampicillin/Sulbactam | Minocycline |
| Azithromycin | Mupirocin |
| Bacitracin Methylene Disalicylate | Neomycin Sulfate, Isoflupredone Acetate, Tetracaine HCl |
| Benzathine Cloxacillin | Neomycin, Polymyxin B & Bacitracin |
| Cefazolin Sodium | Neomycin, Polymyxin B & Dexamethasone |
| Cefotaxime Sodium | Neomycin, Thiabendazole, and Dexamethasone |
| Cefovecin Sodium | Nitrofurantoin Macrocrystals |
| Cefoxitin Sodium | Nitrofurazone |
| Cefpodoxime Proxetil | Nystatin, Neomycin Sulfate, Thiostrepton, Triamcinolone Acetonide |
| Ceftazidime | Ofloxacin |
| Ceftiofur Crystalline Free Acid | Orbifloxacin |
| Ceftiofur Hydrochloride | Orbifloxacin/Mometasone Furoate |
| Ceftiofur Sodium | Monohydrate/Posaconazole |
| Cephalexin | Oxytetracycline Hydrochloride, Polymyxin B (Ointment) |
| Cephapirin Benzathine | Oxytetracycline |
| Cephapirin Sodium | Penicillin G Benzathine and Penicillin G Procaine |
| Chloramphenicol | Penicillin G Potassium |
| Ciprofloxacin | Penicillin G Procaine |
| Clarithromycin | Piperacillin and Tazobactam |
| Clindamycin | Polymyxin B Sulfate |
| Doxycycline | Polymyxin B Sulfate, Miconazole Nitrate, Prednisolone Acetate |
| Enrofloxacin | Pradofloxacin |
| Enrofloxacin/Silver Sulfadiazine | Rifampin |
| Erythromycin (ophthalmic) | Silver sulfadiazine |
| Erythromycin Lactobionate | Sulfadiazine/Trimethoprim |
| Florfenicol | Sulfadimethoxine |
| Gentamicin Sulfate (ophthalmic) | Sulfamethoxazole/Trimethoprim |
| Gentamicin Sulfate and Betamethasone Acetate | Tetracycline |
| Gentamicin Sulfate and Betamethasone Valerate | Tilmicosin Phosphate |
| Gentamicin Sulfate | Tobramycin |
| Gentamicin Sulfate, Mometasone Furoate, Clotrimazole | Tulathromycin |
| Hydrocortisone/Miconazole Nitrate/Gentamicin Sulfate | Tylosin |
| Imipenem/Cilastatin | Vancomycin |
| Lincomycin Hydrochloride |  |

Supplementary Data 2. Specific diagnoses included for each indication for antimicrobial prescription

| Indication | Specific Diagnoses |
| --- | --- |
| Gastrointestinal | Foreign body, gastroenteritis (hemorrhagic, non-hemorrhagic, parvoviral, feline panleukopenia, clostridium, diarrhea, campylobacter), gastric ulceration, hepatic disease (Acute liver injury, cholangiohepatitis), biliary disease including extra hepatic biliary duct obstruction, pancreatitis, rectal diseases (prolapse, wounds, rectal foreign body), splenic torsion |
| EENT | Corneal disease (ulceration, perforation, inflammation), ruptured globe, enucleation, retrobulbar mass with exophthalmos, periocular wounds, third eyelid lacerations, oral diseases (including abscess, fistula, ulceration, severe dental disease, complications of dental prophylaxis and tooth extractions, fracture, TMJ luxation, sublingual inflammation/granuloma), aural hematoma, otitis interna/media/externa, vestibular syndrome, epistaxis |
| Endocrine | Diabetic ketoacidosis (concurrent diseases included pancreatitis, pheochromocytoma and infected mass), hyperosmolar non-ketotic diabetes mellitus, hypoglycemia with regurgitation |
| Reproduction | Pyometra, dystocia, mastitis |
| Musculoskeletal | Tendonitis, open fracture, osteomyelitis, bilateral trapezius hypertrophy with hyperesthesia |
| Neoplasia | Facial swelling/mass, undiagnosed abdominal mass, hypoglycemia with concurrent neoplasia and purulent discharge, abscessed mass, mesenteric mass, oral mass with necrosis, ulcerated mast cell tumor, metastatic neoplasia with MDR wound |
| Neurologic | Cuterebra, undiagnosed neurologic disease with cranial nerve deficits, seizures, encephalitis, tetanus, meningioma |
| Respiratory | Unspecified respiratory disease, unspecified mass in respiratory system with concurrent respiratory signs, aspiration pneumonia, canine infectious respiratory disease complex, upper respiratory infection, pyothorax, abscess in respiratory system, pneumonia (unspecified and bacterial), feline asthma, non-cardiogenic pulmonary edema, collapsing trachea with tracheitis, pulmonary hypertension, chronic bronchitis, pyogranulomatous laryngitis, suspect nasal mass with obstruction of nares, suspect lymphoma with respiratory signs |
| Sepsis | Gallbladder mucocele, septic peritonitis, abscess (liver, pancreatic), dystocia complications, wounds/necrotic wounds, septic pericarditis, complications of surgical dehiscence |
| Skin | Wounds/lacerations, bite wounds, surgical site infections, skin infection (pyotraumatic dermatitis), panniculitis, abscesses, cellulitis |
| Tick-borne | Lyme, anaplasma, immune mediated thrombocytopenia and/or anemia with suspected tick-borne cause |
| Urinary | Urinary tract infection, chronic kidney disease, acute kidney injury, uroabdomen, prostatitis, pyelonephritis |
